# Supplementary material for: Grain-boundary-rich polycrystalline monolayer WS2 film for attomolar-level Hg2+ sensors
Source: Nat Commun. 2021 Jun 23;12:3870. doi: 10.1038/s41467-021-24254-x (PMC8222231; doi:10.1038/s41467-021-24254-x)
Supplement: Supplementary file 1 — Supplementary Information [file 41467_2021_24254_MOESM1_ESM.pdf]

Grain-boundary-rich polycrystalline monolayer WS_2 film for attomolar-level Hg^{2+} sensors

Lixuan Liu[1,2,#], Kun Ye[1,#], Changqing Lin[3], Zhiyan Jia[4,5], Tianyu Xue[1,*], Anmin Nie[1,*], Yingchun Cheng[3], JianYong Xiang[1], CongPu Mu[1], Bochong Wang[1], Fusheng Wen[1], Kun Zhai[1], Zhisheng Zhao[1], Yongji Gong[2,*], Zhongyuan Liu[1,*], Yongjun Tian[1]

[1]Center for High Pressure Science, State Key Lab of Metastable Materials Science and Technology, Yanshan University, Qinhuangdao 066004, People's Republic of China

[2]School of Materials Science and Engineering, Beihang University, Beijing 100191, People's Republic of China

[3]Key Laboratory of Flexible Electronics & Institute of Advanced Materials, Jiangsu National Synergetic Innovation Center for Advanced Materials, Nanjing Tech University, 30 South Puzhu Road, Nanjing 211816, China

[4]International Collaborative Laboratory of 2D Materials for Optoelectronic Science and Technology of Ministry of Education, Engineering Technology Research Center for 2D Materials Information Functional Devices and Systems of Guangdong Province, Institute of Microscale Optoelectronics, Shenzhen University, Shenzhen 518060, People's Republic of China

[5]International Iberian Nanotechnology Laboratory (INL), Avenida Mestre José Veiga, Braga, 4715-330, Portugal

Correspondence should be addressed to T.Y.X. (tyxue@ysu.edu.cn), A.M.N. (e-mail: anmin@ysu.edu.cn), Y.J.G. (e-mail: yongjigong@buaa.edu.cn) or Z.Y.L. (e-mail: liuzy0319@ysu.edu.cn).

Supplementary Discussion

The density of GBs and its dependence on grain sizes. The density of GBs in the CVD-grown polycrystalline 1L WS_2 film can be here defined as the total GB length per unit surface area. The density of GBs is highly dependent on the surface density of 1L WS_2 grains, i.e. the grain sizes and their distribution. With H_2S gas being chosen as the S source in the CVD growth, the nanoscale 1L WS_2 crystals of equilateral triangular shape and high surface density are initially grown on the substrate with uniform distribution, as shown in the SEM images of **Supplementary Figure 1**. The nanoscale sizes of 1L WS_2 crystals are also relatively uniform, lying in a narrow distribution around ~30 nm. In the CVD growth of polycrystalline 1L WS_2 film on a SiO_2 (300 nm)/Si substrate, we have been able to control the growth of 1L WS_2 grains in the nanoscale sizes ranging from ~10 to several tens of nm, and the 1L WS_2 grains have been scarcely observed to grow in sizes larger than 100 nm. The smaller sizes of 1L WS_2 grains imply the higher density of GBs or the longer total GB length per unit surface area, which is able to provide the more structural defects as active sites for capture of analyte and thus the higher sensing performance of the GB-based sensor. The microscale or larger 1L WS_2 grains are actually not desirable for the great enhancement of sensor performance, because the larger grain sizes mean the lower density of GBs.

On a simplified assumption that the CVD-grown GB-rich polycrystalline 1L WS_2 film was formed by the equilateral triangular 1L WS_2 crystals of the same

size stitched together along the edges of neighboring grains, the grain size dependence of the GB density can be well understood, though the assumed simple case is experimentally impossible. For an equilateral triangular 1L WS_2 crystal with the lateral length of ℓ, its surface area of $\mathcal{S}$ and total edge length of $\mathcal{L}$ are given by $\mathcal{S} = \sqrt{3}\ell^2/4$ and $\mathcal{L} = 3\ell$, respectively. The surface density $\mathcal{N}$ or the number of 1L WS_2 crystals per unit surface area is determined by $\mathcal{N} = 1/\mathcal{S} = 4/\sqrt{3}\ell^2$, and the total edge length per unit area is given by $\mathcal{D} = \mathcal{N} \cdot 3\ell = 4\sqrt{3}/\ell$. Since the GBs are assumed to be formed by the stitched edges of neighboring 1L WS_2 crystals, the density of GBs can be expressed as $\mathcal{D}_{\mathrm{GB}} = 2\sqrt{3}/\ell$, which is inversely proportional to the lateral length of ℓ. As shown in inset of **Supplementary Figure 1**, the shorter lateral length of ℓ means the higher density of GBs, but the sharply increased density of GBs occurs only when the lateral length of ℓ or the grain size is decreased to the nanoscale range. Thereby, in order to hugely increase the density of GBs, the grains are required to be grown in the nanoscale sizes. With respect to the value of $\mathcal{D}_{\mathrm{GB}}$ at the microscale lateral length, for example, ℓ = 5 μm, the value of $\mathcal{D}_{\mathrm{GB}}$ at the nanoscale lateral length of ℓ = 40 nm is hugely increased by more than 10^4 %. For a polycrystalline film formed with the 1L WS_2 grains of microscale sizes, the slightly increased density of GBs is not very helpful for effective enhancement of the sensing performance of GB-based sensor in comparison to a single 1L WS_2 crystal. Moreover, for the microscale grains in a wide size distribution, large fluctuation is inevitably induced in the density or

distribution of GBs across the film, leading to the inconsistent sensing performances from different areas of the film due to limited spot size of detection light (170 μm in diameter for our SPR equipment). In the CVD-grown GB-rich polycrystalline 1L WS_2 film formed with the nanoscale 1L WS_2 grains, the size distribution is also constrained in the nanoscale range, and only minute local fluctuation exists in the density of GBs across the film. Considering the spot size of detection light is 170 μm, which is 3 orders larger than nanoscale grains, the density and distribution of GBs within the detection range is thus pretty uniform for different areas, guaranteeing consistent sensing performances of the GB-based SPR sensor from different areas of the film (**Supplementary Figure 2**). Meanwhile, in our CVD-grown polycrystalline 1L WS_2 film by using the S powders as S source (**Supplementary Figure 3**), the film exhibits the large grains in a wide size distribution from several tens to ~200 μm. The large 1L WS_2 grains and the wide size distribution inevitably lead to the large local fluctuation in the density or distribution of GBs across the film. The large local fluctuation in density or distribution of GBs is found to induce the inconsistent sensor performances in Hg^{2+} detection from the different areas of the film, as shown in **Supplementary Figure 4**.

Detection accuracy of GB-based SPR sensor. Detection accuracy of a SPR sensor is defined as $1/W_{FWHM}$, where W_{FWHM} is the full width at half maximum of SPR spectrum.[1] The values of Da were extracted from SPR spectra of the GB-rich 1L WS_2 film, which were collected during the sensor responses to the

Hg^{2+} solutions from 10^{-18} to 10^{-11} M. The accuracy of ~0.4 was determined, which is better than that of graphene-based SPR sensor (~0.2, 680 nm laser light)[2]

Supplementary Figures

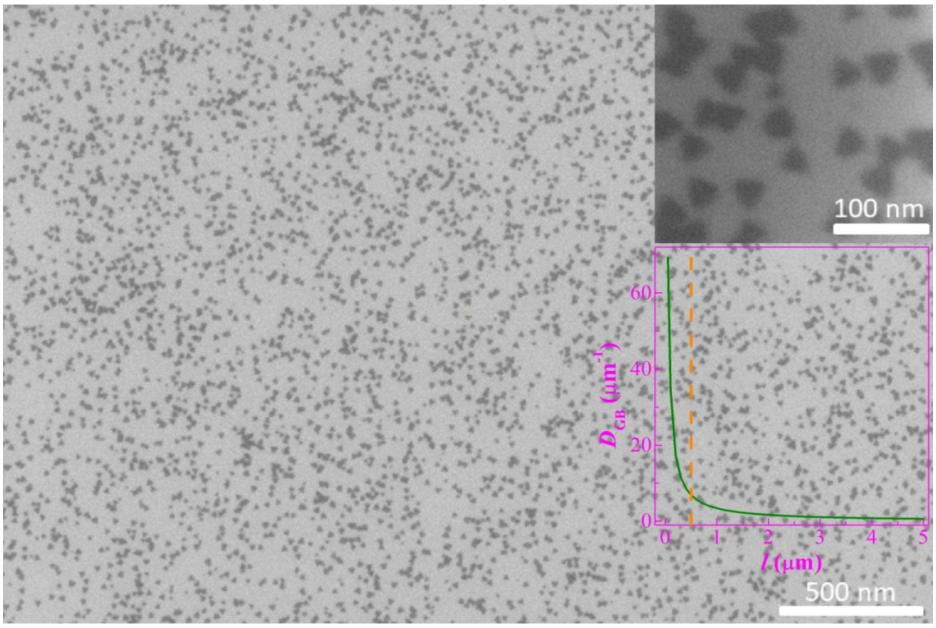

Supplementary Figure 1. SEM image of the initially grown 1L WS_2 nanocrystals. The initially grown 1L WS_2 nanocrystals of equilateral triangular shape and high surface density on a SiO_2 (300 nm)/Si substrate, which were intercepted by intentionally stopping the CVD growth process of the GB-rich polycrystalline 1L WS_2 film. Inset on top is the high-magnification SEM image, and inset on bottom gives the calculated density $\mathcal{D}_{\mathrm{GB}}$ of GBs as a function of lateral length ℓ.

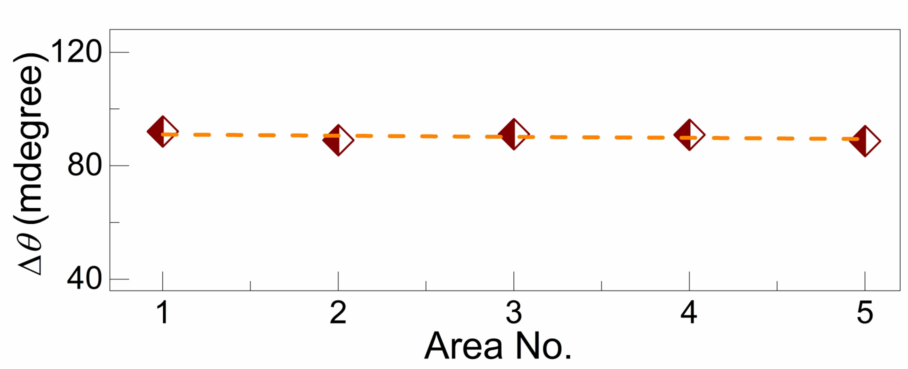

Supplementary Figure 2. Determined resonance angle shifts of Δ*θ* from 5 different areas on the CVD-grown GB-rich polycrystalline 1L WS_2 film. The consistent values of Δ*θ* indicate the desirable growth of nanoscale grains for consistent detection of Hg^{2+} from the different locations of the film because the spot size of detection light (170 μm in diameter) is much greater than nanoscale grain dimensions.

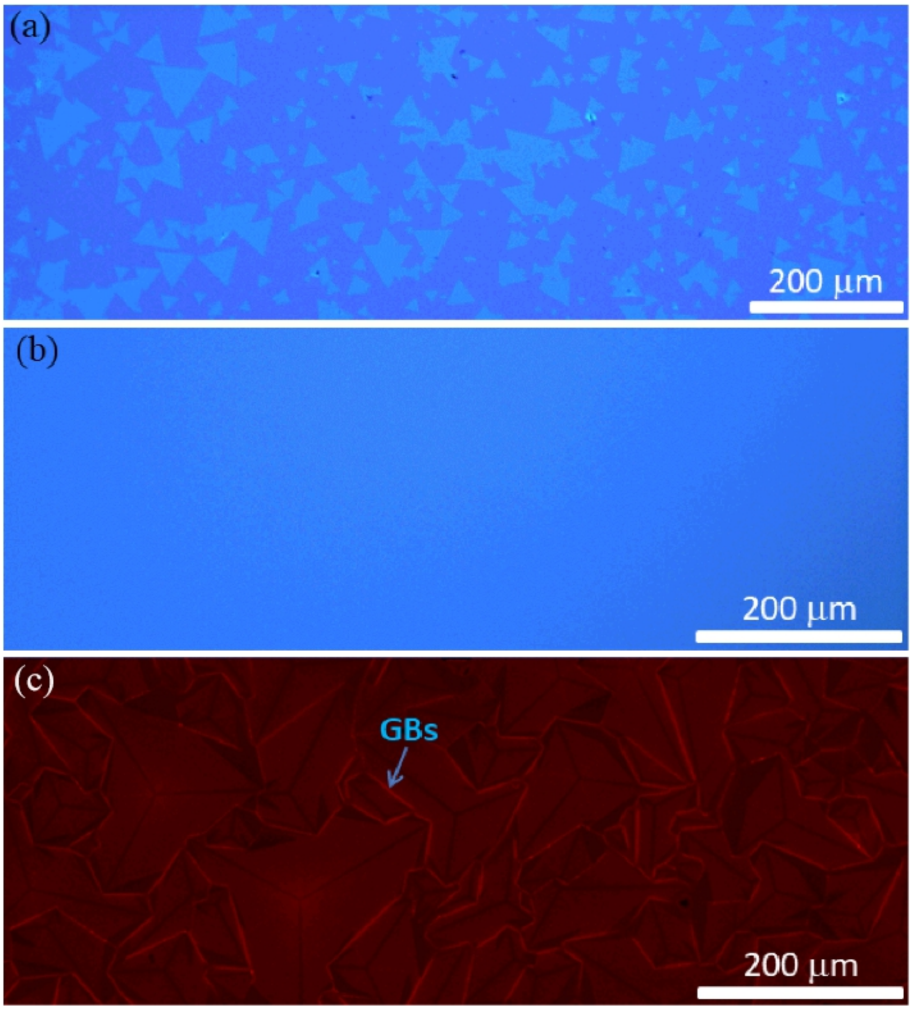

Supplementary Figure 3. 1L WS_2 film with microscale grains by using the S powder as S source. a OM image of the microscale 1L WS_2 crystals grown on SiO_2 (300 nm)/Si substrate, which were intercepted by intentionally stopping the CVD growth process of polycrystalline 1L WS_2 film in the early stage. The S powder was used as S source in the CVD growth. **b** OM image of the CVD-grown polycrystalline 1L WS_2 film. **c** Corresponding FL image. In the FL image, the FL-enhanced stripes come from the GBs.[3] As revealed in the FL image, the 1L WS_2 grains lie in a large size distribution from several tens to ~200 μm, and thus the quite non-uniform distribution of GBs leads to large local fluctuation in the density of GBs across the film.

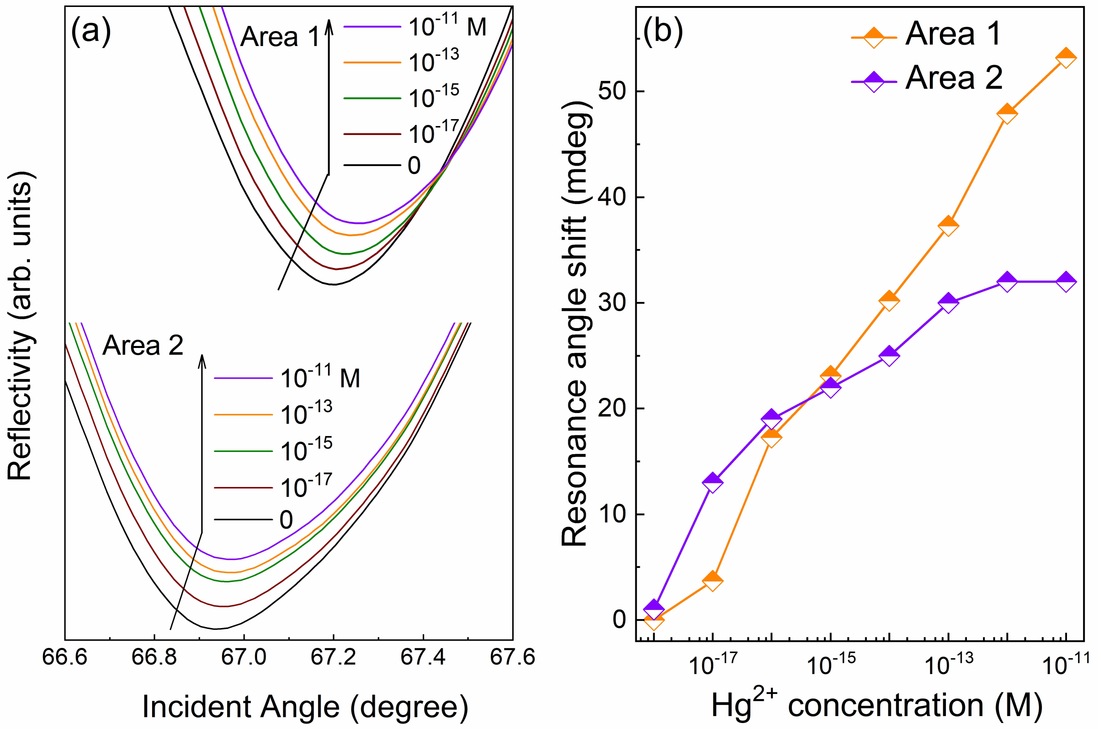

Supplementary Figure 4. Sensing performances of SPR sensor based on 1L WS_2 film with microscale grains. The sensor was fabricated with the CVD-grown polycrystalline 1L WS_2 film as shown in **Supplementary Figure 3b**. **a** SPR spectra taken from two different areas on the polycrystalline 1L WS_2 film as sensing material. **b** Resonance angle shifts of $\Delta\theta$ as a function of Hg^{2+} concentration, which were determined from the SPR spectra in **a**. Obviously, different areas on the film generate the inconsistent sensor responses due to large fluctuation in the density of GBs across the film.

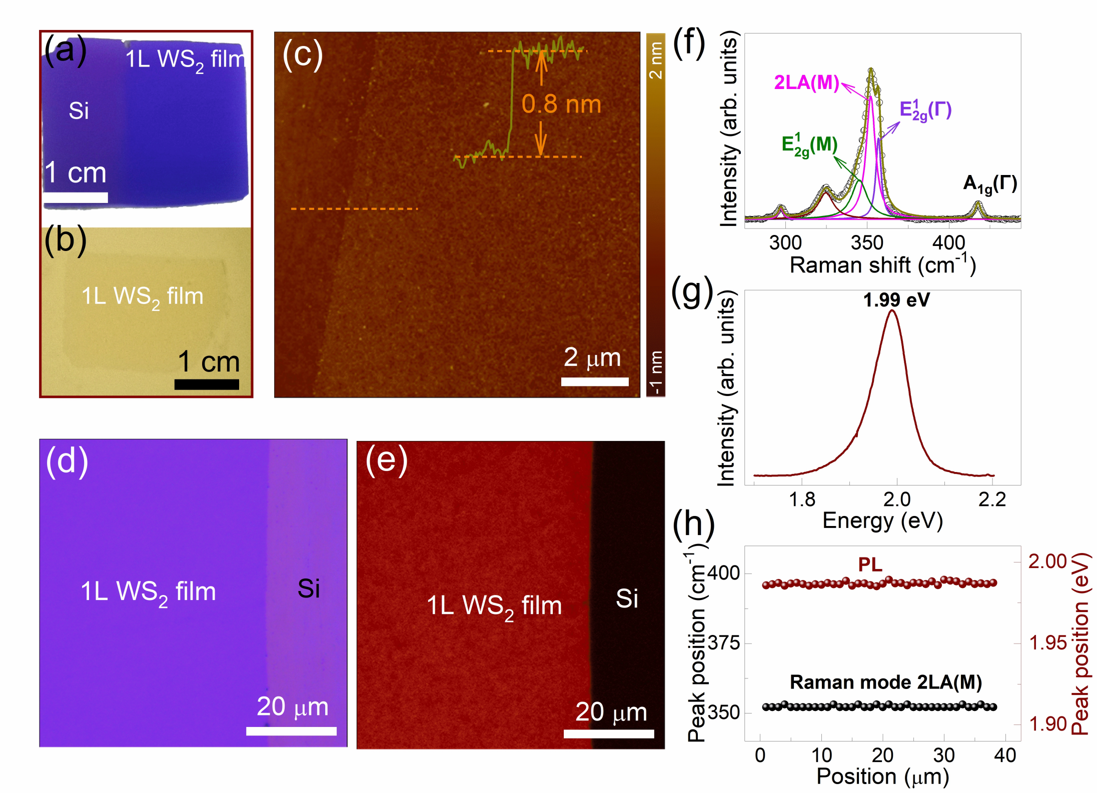

Supplementary Figure 5. Characterization of CVD-grown GB-rich polycrystalline 1L WS_2 films on SiO_2 (300 nm)/Si substrates. a,b Optical photographs of as-grown 1L WS_2 film on SiO_2 (300 nm)/Si substrate and the transferred one on to Au (47 nm) coated cover glass substrate. **c-e** AFM, OM and corresponding FL images of as-grown 1L WS_2 film on SiO_2 (300 nm)/Si substrate. A scratch was made on the 1L WS_2 film to differentiate between the film and Si substrate due to their contrast difference. Inset in c is a height profile taken along the dash line, showing the thickness of 0.8 nm. **f,g** Raman and PL spectra of the 1L WS_2 film. **h** Raman and PL line scans across the 1L WS_2 film. All Raman and PL measurements were performed by using 532 nm laser light excitation.

In OM image of **Supplementary Figure 5d**, the 1L WS_2 film can be distinctly recognized due to the difference in optical contrasts between the film and bare SiO_2/Si substrate. The as-synthesized 1L WS_2 film exhibits uniform optical contrast, signifying an even and high-quality continuous film. In the FL image of **Supplementary Figure 5e**, the uniformity of FL intensity across the film implies the monolayer nature and high quality of the as-synthesized 1L WS_2 film. As displayed in **Supplementary Figure 5f and g**, the observed Raman modes of E^1_{2g}(M) (345.1 cm^{-1}), *2LA*(M) (352.15 cm^{-1}), E^1_{2g}(Γ) (356.9 cm^{-1}) and A_{1g} (Γ) (417.6 cm^{-1}), as well as the single photon emission at ~ 1.99 eV are in good agreement with previous spectral results regarding CVD-grown 1L WS_2.[4-6] The Raman intensity ratio of $I_{2LA(M)}/I_{A1g(\Gamma)}$ is determined to be 7.6, which is a characteristic of monolayer thickness, as $I_{2LA(M)}/I_{A1g(\Gamma)}$ is larger than 2 only for monolayer WS_2.[6] Raman and PL line scans in **Supplementary Figure 5h** demonstrate the high consistency in peak position and intensity at different film locations, confirming the high quality of the as-synthesized polycrystalline 1L WS_2 film.

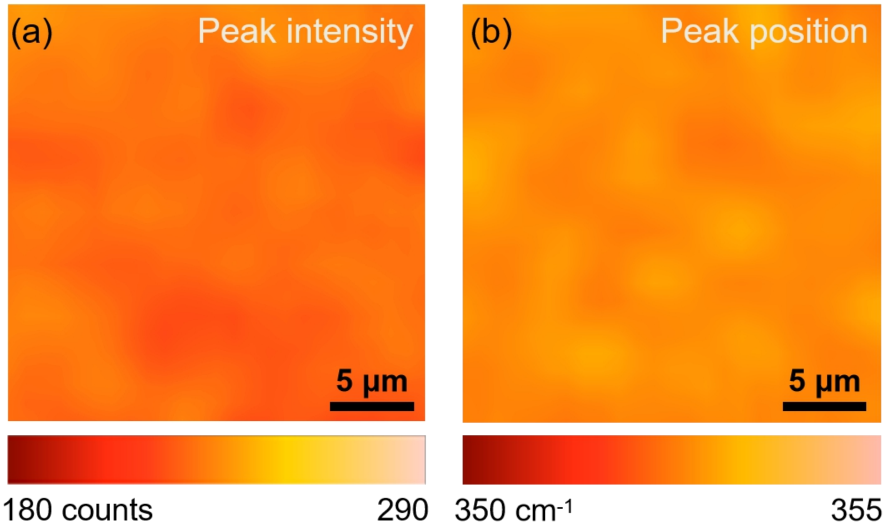

Supplementary Figure 6. Raman *2LA*(M) mapping images of as-synthesized GB-rich 1L WS_2 film. a Raman mapping image of 2LA(M) peak intensity. **b** Raman mapping image of 2LA(M) peak position. 532 nm laser was used for excitation. Raman mapping images show the high consistency in peak position and intensity at different film locations.

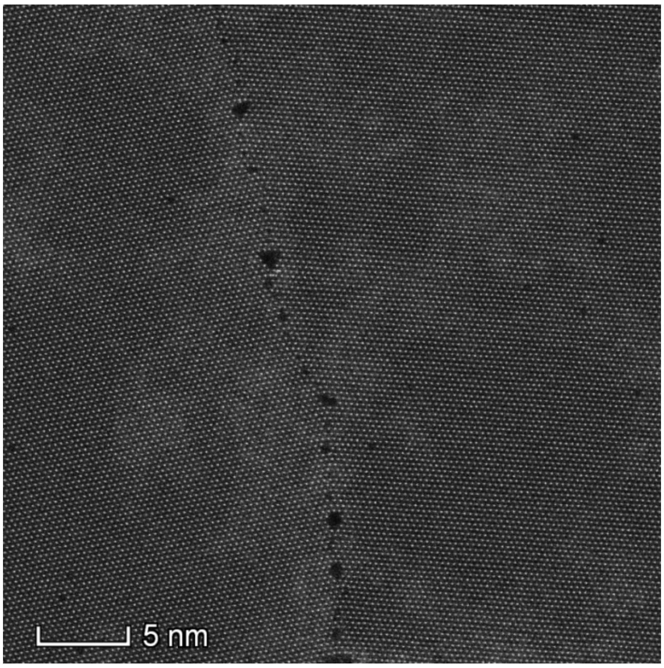

Supplementary Figure 7. Atomic scale HAADF-STEM image. Typical GB in the GB-rich polycrystalline 1L WS_2 films.

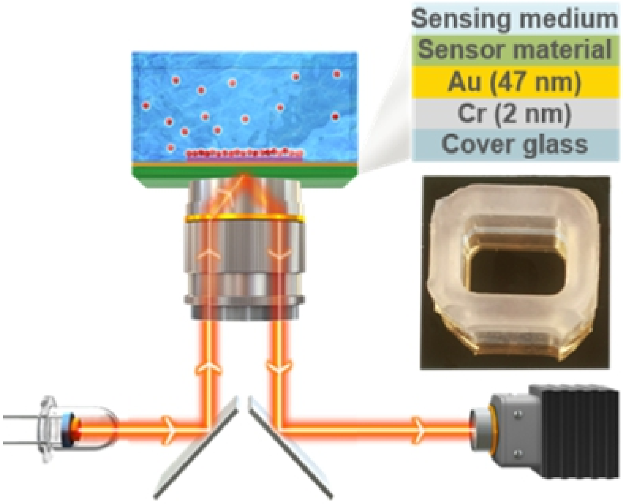

Supplementary Figure 8. Schematic of the SPR setup. Schematic illustration of the SPR imaging setup and the sensor device based on GB-rich 1L WS_2 film.

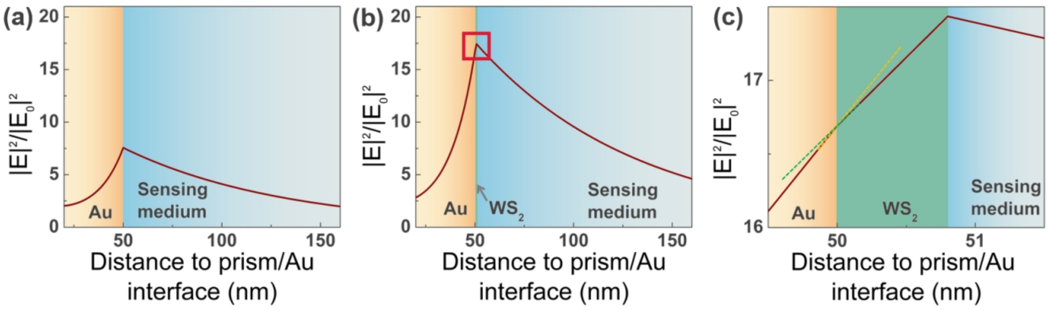

Supplementary Figure 9. The electric field distribution of the proposed SPR sensor based on polycrystalline 1L WS_2 film. a Simulated electric field distributions between bare 50 nm Au film and sensing medium. **b** Simulated electric field distributions between polycrystalline 1L WS_2 film-covered 50 nm Au and sensing medium. **c** is the zoom in of the red square area in **b**.

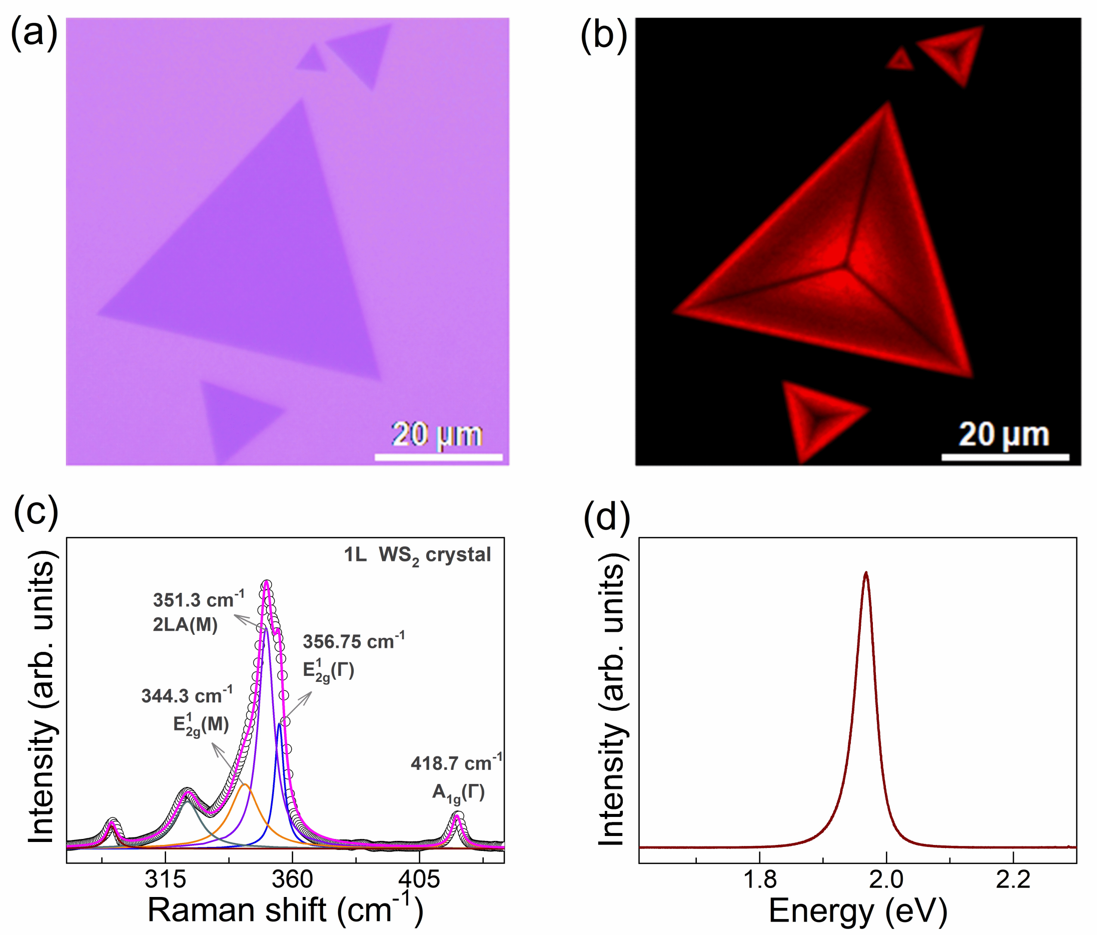

Supplementary Figure 10. Characterization of CVD-grown 1L WS_2 single crystals. a-d OM, corresponding FL images, Raman and PL spectra of CVD-grown 1L WS_2 single crystals on SiO_2 (300 nm)/Si substrate. In the FL image of **Supplementary Figure 10b**, the observed strong FL signal indicates the monolayer nature of CVD-grown WS_2 crystals. The measured Raman and PL spectra in **Supplementary Figure 10c** and **d**, respectively, are consistent with the previously reported ones for 1L WS_2 crystals.[6]

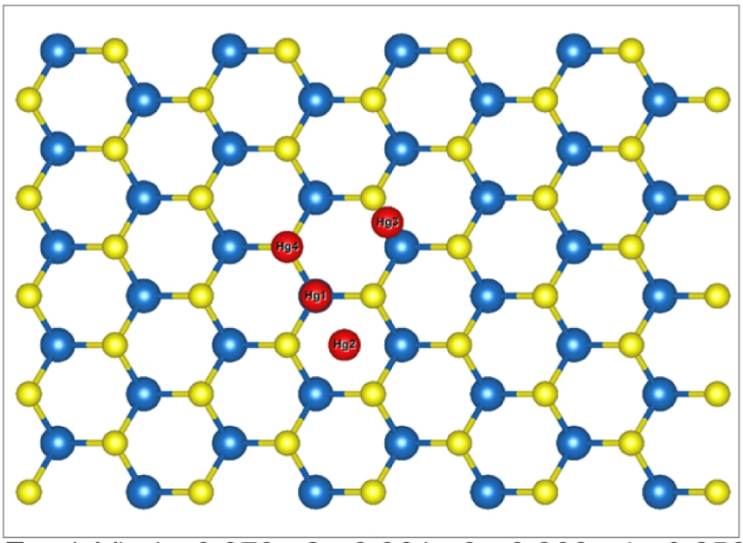

E_{ads} (eV): 1. -0.379, 2. -0.361, 3. -0.308, 4. -0.256

Supplementary Figure 11. DFT calculations about different adsorption positions of Hg^{2+} away from the GBs in 1L WS_2 film labeled out with number 1-4. Yellow, blue and red spheres represent S, W and Hg atoms, respectively. The calculated adsorption energies of E_{ads} at four different positions 1-4 are listed on the bottom.

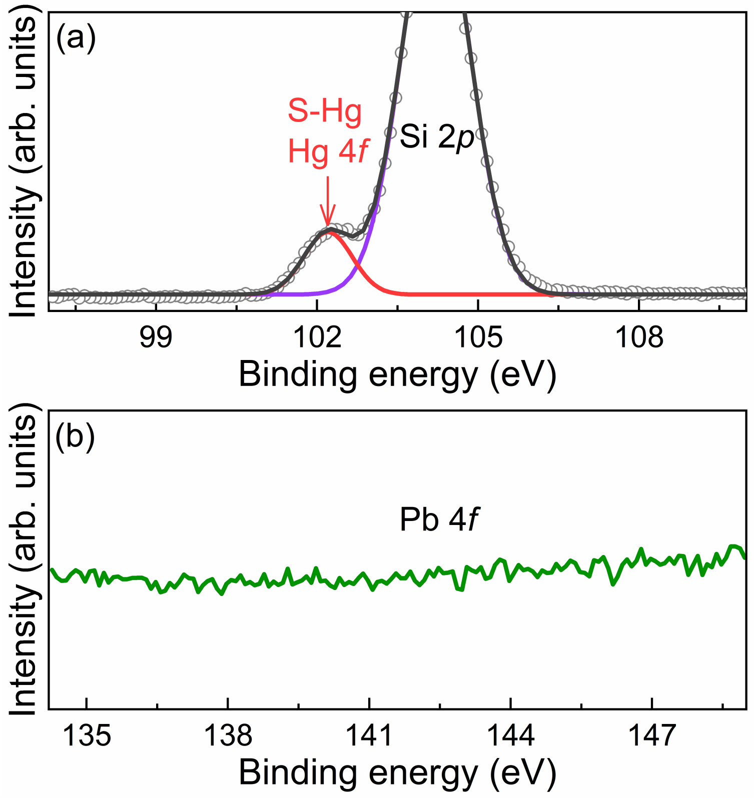

Supplementary Figure 12. XPS characterization. a,b XPS spectra of Hg 4*f* and Pb 4*f* core levels taken from the GB-rich polycrystalline 1L WS_2 film as sensing material after ion detection in the mixed Hg^{2+} and Pb^{2+} solutions, in which Hg^{2+} concentration is 10^{-15} M and Pb^{2+} concentration is 10^{-12} M.

For the Pb–S bonds, the Pb $4f_{7/2}$ and Pb $4f_{5/2}$ peaks occur at the binding energies of ~137.5 and ~142.9 eV, respectively.[7] In the of Hg 4*f* core level XPS spectrum **(Supplementary Figure 12a)**, the distinct peak around 102 eV is recognized to come from Hg 4*f* core level. In the of Pb 4*f* core level XPS spectrum **(Supplementary Figure 12b)**, however, no sign of peak is observable around the binding energies of ~137.5 and ~142.9 eV on the background in the detection limit. Therefore, for Hg^{2+} detection of GB-based SPR sensor in the mixed Hg^{2+} and Pb^{2+} solutions, the interference to the

sensor response from the coexisting Pb^{2+} ions can be neglected. The absorption of Hg^{2+} ions on GBs are preferential in the mixed solution of coexisting Hg^{2+} and Pb^{2+} ions.

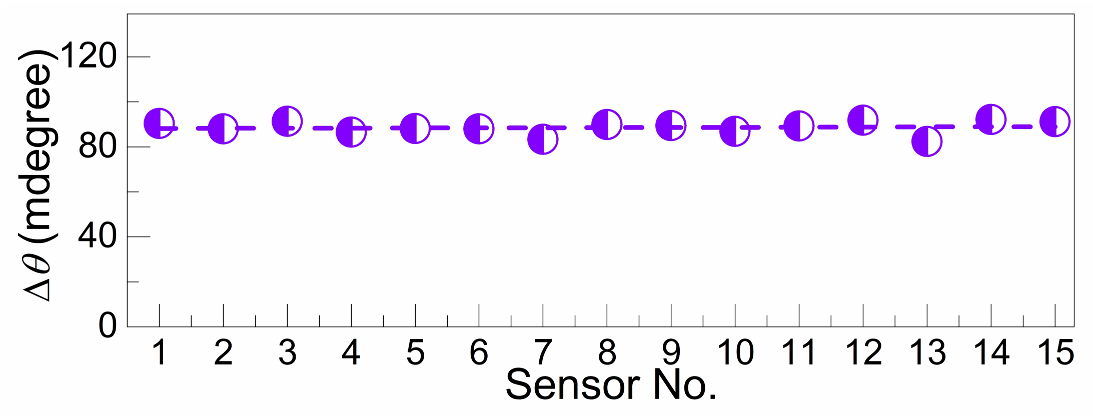

Supplementary Figure 13. The repeatability of the GB-based SPR sensor. Resonance angle shifts of $\Delta\theta$, which were determined from the responses of 15 GB-based SPR sensors to the 10^{-15} M Hg^{2+} solution. These sensors were fabricated with GB-rich polycrystalline 1L WS_2 films from 15 batches grown at different dates. The consistency in values of $\Delta\theta$ and small standard deviation indicates the high repeatability($\Delta\theta = 89.28 \pm 2.89$ mdeg).

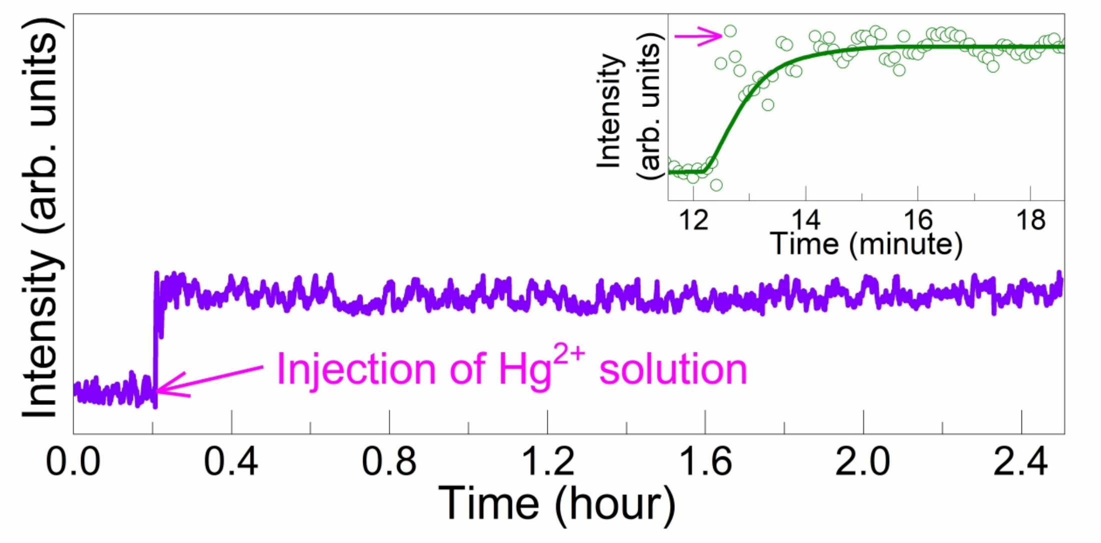

Supplementary Figure 14. Response time of the GB-based SPR sensor. Response of one GB-based SPR sensor to 10^{-15} M Hg^{2+} solution monitored by the Kinetic curve for more than 2 hours. Inset is the amplified Kinetic curve to show the initial response. The arrow-pointed jump is induced by injection of Hg^{2+} solution.

In Hg^{2+} detection, the sensor response can be monitored via the Kinetic curve, and generally, it requires a response time of several minutes to reach the dynamical equilibrium for signal collection, similar to any other SPR sensor.[8-10] After the dynamical equilibrium, the steady sensor response was continuously monitored for more than 2 hours, and no observation of any abrupt change indicates the high sensor stability in Hg^{2+} solution.

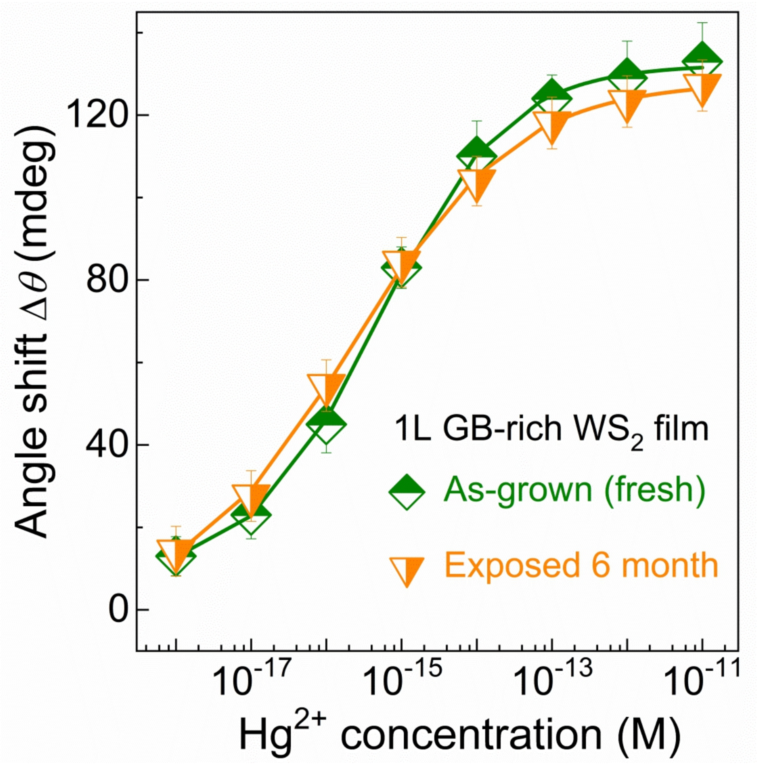

Supplementary Figure 15. Environmental stability of GB-rich 1L WS_2 film. Resonance angle shifts of $\Delta\theta$ determined from SPR spectra of freshly grown and 6-month exposed GB-rich 1L WS_2 film at Hg^{2+} concentrations ranging from 10^{-18} M to 10^{-11} M. All error bars is the standard deviation of SPR angle shift from five repeated measurements.

To examine the environmental stability of GB-rich polycrystalline 1L WS_2 film, the performance of a GB-based sensor after 6-month exposure under ambient conditions was examined. Notably, the sensor was treated for 10 minutes at 260 °C in a tubular furnace under the protecting flow of mixed H_2 (1%) and Ar gas for removal of the absorbed contaminants on GBs, and then its responses to Hg^{2+} solutions were checked. The determined resonance angle shifts of freshly grown and 6-month ambiently exposed 1L GB-rich WS_2 film are presented in **Supplementary Figure 15** for comparison. After 6-month

exposure to air, the sensor still exhibits comparable sensor performance, with only slight deterioration at higher concentrations, to that of the fresh as-grown GB-rich 1L WS_2 film. These observations indicate the excellent environmental stability of 1L GB-rich WS_2 film for GB-based sensor.

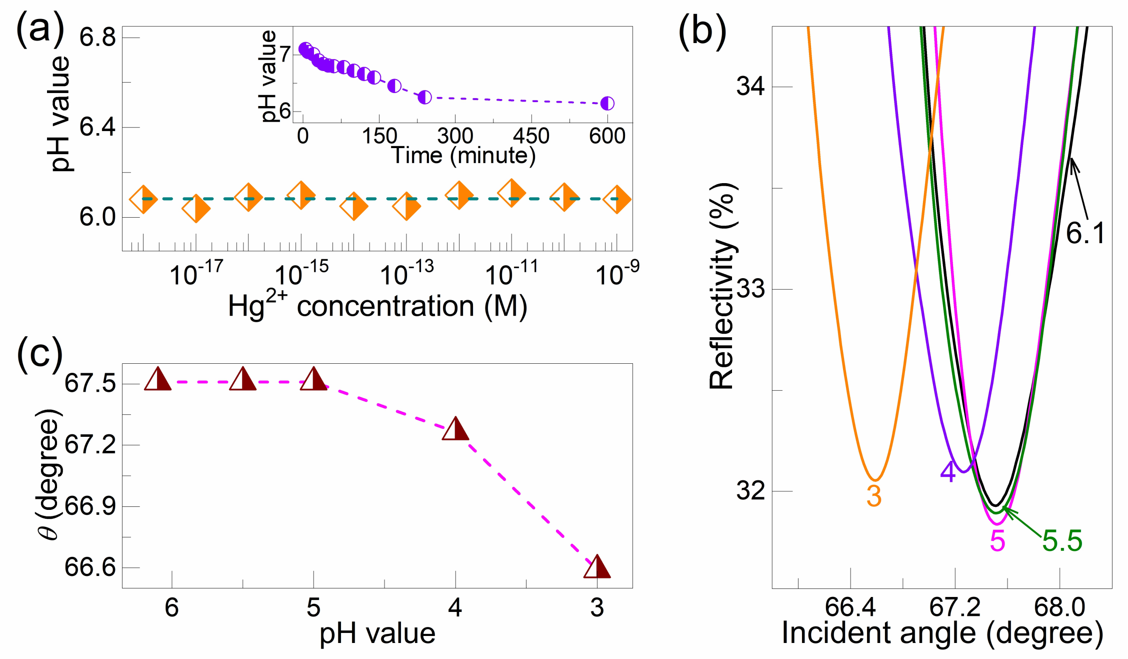

Supplementary Figure 16. Sensor response to acidified water. a pH values in the prepared Hg^{2+} solutions, which slightly fluctuate around 6.1. Inset is the time-dependent pH value of ultrapure water after preparation. The pH value of as-prepared ultrapure water is around 7, but it decreases with time to a stable value of ~6 under exposure to air. No acidification of water was done in the preparation of Hg^{2+} solutions. **b** SPR spectra of GB-based SPR sensor in a series of the acidified water. Acidification of ultrapure water to the pH value ranging from 6.1 to 3 was performed by adding nitric acid (HNO_3). **c**

Corresponding resonance angle shifts of $\Delta\theta$ extracted from the SPR spectra in **b.**

Based on the SPR spectra in (**Supplementary Figure 16b**) and the corresponding values of $\Delta\theta$ in (**Supplementary Figure 16c**), for the pH value of larger than 5, no interference to the sensor response from acid is observable, but the interference from acid becomes observable when the pH value becomes lower than 5. With decreasing pH value, the interference from acid becomes stronger. Thereby, in Hg^{2+} detection, the pH value of detected ion solution were carefully checked to rule out interference due to pH. As shown in **Supplementary Figure 16a**, pH values of prepared Hg^{2+} solutions are around 6.1, at which pH does not impact sensor response.

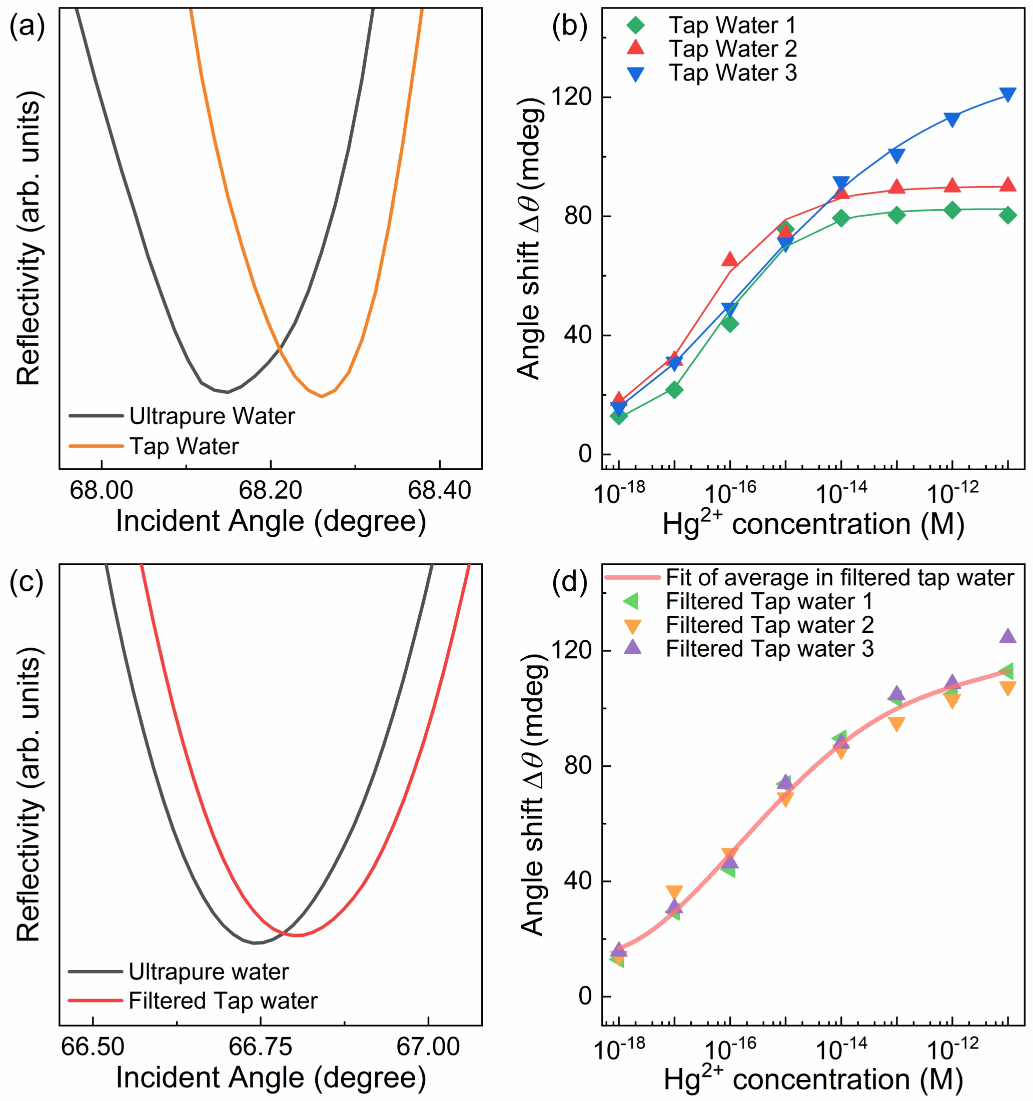

Supplementary Figure 17. Hg^{2+} detection of the GB-based sensor in tap water matrix. a SPR spectra of GB-based SPR sensors in ultrapure water and tap water without additional filtration for comparison. **b** Resonance angle shifts of $\Delta\theta$ extracted from the SPR spectra of 3 GB-based sensors at Hg^{2+} concentrations ranging from 10^{-18} M to 10^{-11} M in the tap water matrix without additional filtration. **c** SPR spectra of GB-based SPR sensors in ultrapure water and filtered tap water using filter paper for comparison. **d** Resonance angle shifts of $\Delta\theta$ extracted from the SPR spectra of 3 GB-based sensors at

Hg^{2+} concentrations ranging from 10^{-18} M to 10^{-11} M in the filtered tap water matrix. The red curve is the fit of the average values of three GB-based sensors.

Hg^{2+} sensing in the tap water matrix using the GB-based sensors was investigated to evaluate its applicability in real case scenarios. The tap water used is up to standards and regulations regarding water safety. The Hg^{2+} solutions were prepared with the tap water after a simple filtration with filter paper, which was first conducted to remove most of the possible insoluble impurities. It is worth noting the difference between SPR responses to the tap water (filtered tap water) and ultrapure water (**Supplementary Figure 17a and c**). However, this observed difference cannot give the conclusion that the tap water is contaminated by Hg^{2+}. Tap water involves the more complex components, including not only the possible metal ions but many other possible types of impurities such as organic impurities, germs, viruses, *etc.* Filtration with filter paper to remove insoluble impurities can help reduce the background signal in tap water matrix and promote better sensor performance (**Supplementary Figure 17b-d**). Though most of the insoluble impurities can be removed by a simple filtration with filter paper, some other complex impurities can still produce the interference to the SPR sensor response. Currently, we cannot exactly identify which impurity produces the different sensor responses to tap water and ultrapure water, but we are able to rule out the possibility of Hg^{2+} contamination in the used tap water.

To rule out the possibility that this shift is caused by Hg^{2+} and to evaluate whether the GB-based sensor can still detect aM level Hg^{2+} in tap water, a series of standard Hg^{2+} solutions (10^{-18}-10^{-11} M) were prepared using the filtered tap water instead of ultrapure water, and the sensor responses to these Hg^{2+} solutions were measured. On the assumption that the possible Hg^{2+} contamination existed in tap water with a concentration higher than the added one of Hg^{2+} ions, the SPR response would be similar to the tap water before the additional introduction of Hg^{2+} ions. **Supplementary Figure 17d** shows the determined resonance angle shift ($\Delta\theta$) as a function of Hg^{2+} concentration in filtered tap water matrix for 3 GB-based sensors, exhibiting the robust and quite consistent device-to-device sensor responses. A clear positive correlation is observed between Hg^{2+} concentration in the filtered tap water (10^{-18} M - 10^{-11} M) and sensor response. For the three tested GB-based sensors, their average value of $\Delta\theta$ can be quantitatively expressed by an empirical formula of $\Delta\theta$ = 19.2*log[Hg^{2+}]+356.7 (R^2 = 0.9820) within the Hg^{2+} concentration range from 10^{-17} M to 10^{-13} M. In comparison to $\Delta\theta$ = 26.7*log[Hg^{2+}]+477.5 (R^2 = 0.9801) obtained in ultrapure water, though the slope of 19.2 in the filtered tap water matrix becomes smaller, it still keeps the same order, and more importantly, the robust response is still produced even at the 1 aM level Hg^{2+} concentration, indicating that the sensitivity of GB-based sensor is not compromised very much in the real tap water matrix and the practical applicability can still be achieved. Based on these results, the

following conclusions can be made: the difference between SPR responses to tap water and ultra-pure water is caused by other impurities rather than Hg^{2+}, and the sensitive Hg^{2+} detection with aM-level LOD can still be achieved in tap water.

The observable resonance angle shift at 1aM, even in tap water matrix, further demonstrates the low LOD of GB-based sensors and shows that GB engineering in WS_2 and other 2D materials is an effective method to design and construct novel sensing materials towards low-limit detection. Notably, by further examination regarding possible sensor interference from complex components in a real matrix, the sensor performance can be further optimized. Nevertheless, the demonstrated sensitive Hg^{2+} sensing in the tap water matrix using the GB-based sensors shows that exploiting GBs of 2D materials in fabrication of ultra-sensitive ion sensors also exhibit great application potential.

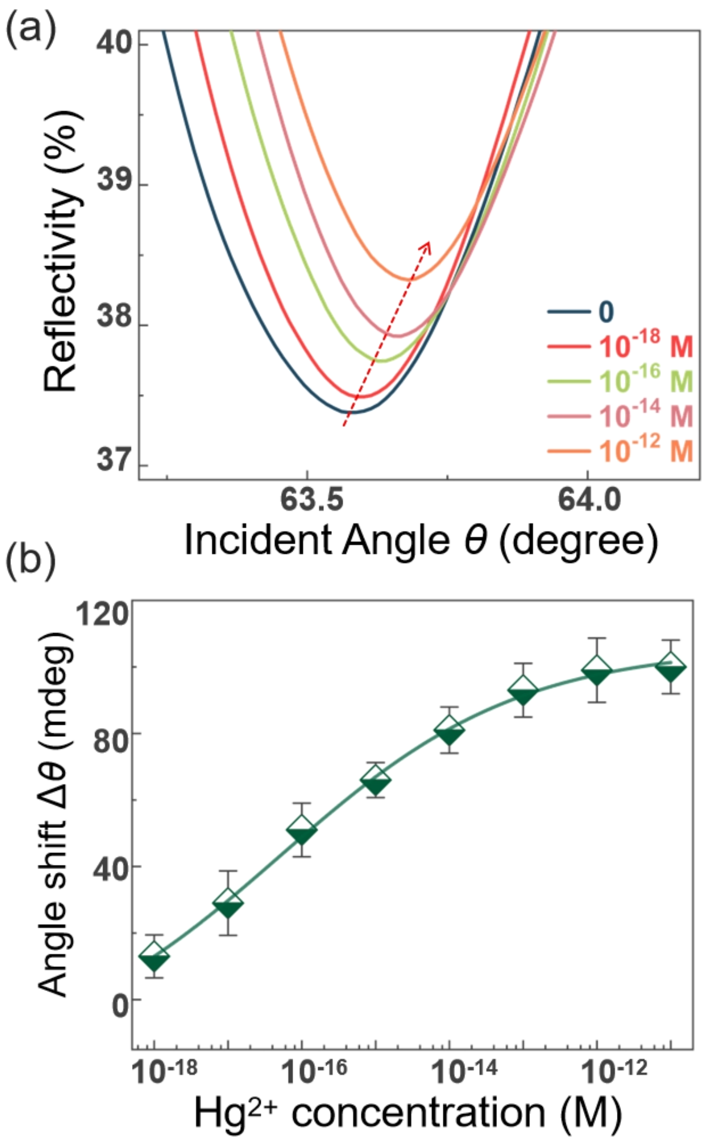

Supplementary Figure 18. Hg^{2+} detection of the SPR sensor based on GB-rich 1L MoS_2 film. a SPR spectra of a SPR sensor based on GB-rich polycrystalline 1L MoS_2 film as sensing material, which were collected during the detection in Hg^{2+} solutions of different concentrations. **b** Corresponding resonance angle shifts of $\Delta\theta$ extracted from the SPR spectra in **a**. All error bars is the standard deviation of SPR angle shift from five repeated measurements.

Supplementary References

1. Wu, L. et al. High-Performance Lossy-Mode Resonance Sensor Based on Few-Layer Black Phosphorus. *J. Phys. Chem. C* **122,** 7368-7373 (2018).
2. Menon, P. S. et al. Multilayer CVD-graphene and MoS_2 ethanol sensing and characterization using Kretschmann-based SPR, *J. Electron. Devices Society* **8,** 1227 (2018).
3. Jia, Z.; et al. Grain wall boundaries in centimeter-scale continuous monolayer WS_2 film grown by chemical vapor deposition. *Nanotechnology* ***29,*** 255705 (2018).
4. Zeng, H. et al. Optical signature of symmetry variations and spin-valley coupling in atomically thin tungsten dichalcogenides. *Sci. Rep*. **3,** 1608 (2013).
5. Gutierrez, H. R. et al. Extraordinary room-temperature photoluminescence in triangular WS_2 monolayers. *Nano Lett*. **13,** 3447-3454 (2013).
6. Berkdemir, A. et al. Identification of individual and few layers of WS_2 using Raman Spectroscopy. *Sci. Rep.* **3,** 1755 (2013).
7. Park, Y. H. et al. Synthesis and Growth Mechanism of Stable Prenucleated (≈0.8 nm Diameter) PbS Quantum Dots by Medium Energy Ion Scattering Spectroscopy. *Materials* **12,** 1109 (2019).
8. Georgiadis, R., Peterlinz, K. P. & Peterson, A. W. Quantitative Measurements and Modeling of Kinetics in Nucleic Acid Monolayer Films Using SPR Spectroscopy. *J. Am. Chem. Soc.* **122,** 3166-3173 (2000).
9. Roy, M. et al. SPR-Measured Dissociation Kinetics of PROTAC Ternary Complexes Influence Target Degradation Rate. *ACS Chem. Biol.* **14,** 361 (2019).
10. Dang, T. et al. Protein binding kinetics quantification via coupled plasmonic-photonic resonance nanosensors in generic microplate reader. *Biosens. Bioelectron.* **142,** 111494 (2019).
11. Xue, T., Qi, K. & Hu, C. Novel SPR sensing platform based on superstructure MoS_2 nanosheets for ultrasensitive detection of mercury ion.

Sens. Actuators, B **284,** 589-594 (2019).

12. Guo, X., Qian, X. & Jia, L. A Highly Selective and Sensitive Fluorescent Chemosensor for Hg^{2+} in Neutral Buffer Aqueous Solution. *J. Am. Chem. Soc.* **126,** 2272-2273 (2004).

13. Du, J. et al. A New Fluorescent Chemodosimeter for Hg^{2+}: Selectivity, Sensitivity, and Resistance to Cys and GSH. *Org. Lett.* **12,** 476-479 (2010).

14. Yang, Y. K., Yook, K. J. & Tae, J. A Rhodamine-Based Fluorescnet and Colorimetric Chemodosimeter for the Rapid Detection of Hg^{2+} Ions in Aqueous Media. *J. Am. Chem. Soc.* **127,** 16760-16761 (2005).

15. Nolan, E. M. & Lippard, S. J. A "Turn-On" Fluorescent Sensor of the Selective Detection of Mercuric Ion in Aqueous Media. *J. Am. Chem. Soc.* **125,** 14270-14270 (2003).

16. Othman, A. B. et al. Calix[4]arene-Based, Hg^{2+}-Induced Intramolecular Fluorescence Resonance Energy Transfer Chemosensor. *J. Org. Chem.* **72,** 7634-7640 (2007).

17. Zhang, X., Xiao, Y. & Qian, X. A ratiometric fluorescent probe based on FRET for imaging Hg^{2+} ions in living cells. *Angew. Chem. Int. Ed. Engl.* **47,** 8025-8029 (2008).

18. Wang, J. & Liu, B. Highly sensitive and selective detection of Hg^{2+} in aqueous solution with mercury-specific DNA and Sybr Green I. *Chem. Commun.* **39,** 4759-4761 (2008).

19. Métivier, R., Leray, I., Lebeau, B. & Valeur, B. A mesoporous silica functionalized by a covalently bound calixarene-based fluoroionophore for selective optical sensing of mercury(ii) in water. *J. Mater. Chem.* **15,** 2965-2973 (2005).

20. Mahato, P. et al. Ganguly, B.; Das, A., Ratiometric detection of Cr^{3+} and Hg^{2+} by a naphthalimide-rhodamine based fluorescent probe. *Inorg. Chem.* **51,** 1769-1777 (2012).

21. Zhou, L., Lin, Y., Huang, Z., Ren, J. & Qu, X. Carbon nanodots as fluorescence probes for rapid, sensitive, and label-free detection of Hg^{2+} and

biothiols in complex matrices. *Chem. Commun.* **48,** 1147-1149 (2012).
22. Zhang, R. & Chen, W. Nitrogen-doped carbon quantum dots: facile synthesis and application as a "turn-off" fluorescent probe for detection of Hg^{2+} ions. *Biosens. Bioelectron.* **55,** 83-90 (2014).
23. Yan, F. et al. Highly photoluminescent carbon dots-based fluorescent chemosensors for sensitive and selective detection of mercury ions and application of imaging in living cells. *Sens. Actuators, B* **192,** 488-495 (2014).
24. Xia, Y. S. & Zhu, C. Q., Use of surface-modified CdTe quantum dots as fluorescent probes in sensing mercury (II). *Talanta* **75,** 215-221 (2008).
25. Wu, L., Liu, H., Liu, T. & Li, C. Nitrogen-doped Carbon Dots: Application of Hg Ions Detection in Rannasangpei. *Chem. Res. Chinese Universities* **35,** 577-580 (2019).
26. Huang, D., Niu, C., Wang, X., Lv, X. & Zeng, G. "Turn-on" fluorescent sensor for Hg^{2+} based on single-stranded DNA functionalized Mn:CdS/ZnS quantum dots and gold nanoparticles by time-gated mode. *Anal. Chem.* **85,** 1164-1170 (2013).
27. Barman, S. & Sadhukhan, M. Facile bulk production of highly blue fluorescent graphitic carbon nitride quantum dots and their application as highly selective and sensitive sensors for the detection of mercuric and iodide ions in aqueous media. *J. Mater. Chem.* **22,** 21832 (2012).
28. Lee, J. S. & Mirkin, C. A. Chip-Based Scanometric Detection of Mercuric Ion Using DNA-Functionalized Gold Nanoparticles. *Anal. Chem.* **80,** 6805-6808 (2008).
29. Xie, J., Zheng, Y. & Ying, J. Y. Highly selective and ultrasensitive detection of Hg^{2+} based on fluorescence quenching of Au nanoclusters by Hg^{2+}-Au^{+} interactions. *Chem. Commun.* **46,** 961-963 (2010).
30. Wu, G. W. et al. Citrate-capped platinum nanoparticle as a smart probe for ultrasensitive mercury sensing. *Anal. Chem.* **86,** 10955-10960 (2014).
31. Wei, H. et al. Lysozyme-stabilized gold fluorescent cluster: Synthesis and application as Hg^{2+} sensor. *Analyst* **135,** 1406-1410 (2010).

32. Wang, Y., Yang, F. & Yang, X. Colorimetric detection of mercury(II) ion using unmodified silver nanoparticles and mercury-specific oligonucleotides. *ACS Appl. Mater. Interfaces* **2,** 339-342 (2010).
33. Wang, Y., Yang, F. & Yang, X. Colorimetric biosensing of mercury(II) ion using unmodified gold nanoparticle probes and thrombin-binding aptamer. *Biosens. Bioelectron.* **25,** 1994-1998 (2010).
34. Shang, L. et al. Microwave-assisted rapid synthesis of luminescent gold nanoclusters for sensing Hg^{2+} in living cells using fluorescence imaging. *Nanoscale* **4,** 4155-4160 (2012).
35. Lu, W. et al. Economical, green synthesis of fluorescent carbon nanoparticles and their use as probes for sensitive and selective detection of mercury(II) ions. *Anal. Chem.* **84,** 5351-53577 (2012).
36. Liu, J., Vellaisamy, K., Yang, G., Leung, C. H. & Ma, D. L. Luminescent turn-on detection of Hg(II) via the quenching of an iridium(III) complex by Hg(II)-mediated silver nanoparticles. *Sci. Rep.* **7,** 3620 (2017).
37. Lin, Z. H. et al. A self-powered triboelectric nanosensor for mercury ion detection. *Angew. Chem. Int. Ed. Engl.* **52,** 5065-5069 (2013).
38. Janani, B. et al. Enhanced SPR signals based on methylenediphosphonic acid functionalized Ag NPs for the detection of Hg(II) in the presence of an antioxidant glutathione. *J. Mol. Liq.* **311,** 113281 (2020).
39. Huang, C.-C., Yang, Z., Lee, K.-H. & Chang, H.-T. Synthesis of Highly Fluorescent Gold Nanoparticles for Sensing Mercury(II). *Angew. Chem.* **119,** 6948-6952 (2007).
40. Huang, C. C. & Chang, H. T. Parameters for selective colorimetric sensing of mercury(II) in aqueous solutions using mercaptopropionic acid-modified gold nanoparticles. *Chem. Commun.* **12,** 1215-1217 (2007).
41. Hu, D., Sheng, Z., Gong, P., Zhang, P. & Cai, L. Highly selective fluorescent sensors for Hg^{2+} based on bovine serum albumin-capped gold nanoclusters. *Analyst* **135,** 1411-1416 (2010).
42. He, S. et al. Design of a gold nanoprobe for rapid and portable mercury

detection with the naked eye. *Chem. Commun.* **40,** 4885-4887 (2008).
43. Guo, Y., Wang, Z., Qu, W., Shao, H. & Jiang, X. Colorimetric detection of mercury, lead and copper ions simultaneously using protein-functionalized gold nanoparticles. *Biosens. Bioelectron.* **26,** 4064-4069 (2011).
44. Guo, C. & Irudayaraj, J. Fluorescent Ag clusters via a protein-directed approach as a Hg(II) ion sensor. *Anal. Chem.* **83,** 2883-2889 (2011).
45. Gong, J., Zhou, T., Song, D., Zhang, L. & Hu, X. Stripping Voltammetric Detection of Mercury(II) Based on a Bimetallic Au-Pt Inorganic-Organic Hybrid Nanocomposite Modified Glassy Carbon Electrode. *Anal. Chem.* **82,** 567–573 (2010).
46. Qi, Y., Xiu, F. R., Yu, G., Huang, L. & Li, B. Simple and rapid chemiluminescence aptasensor for Hg^{2+} in contaminated samples: A new signal amplification mechanism. *Biosens. Bioelectron.* **87,** 439-446 (2017).
47. Zhang, L., Li, T., Li, B., Li, J. & Wang, E. Carbon nanotube-DNA hybrid fluorescent sensor for sensitive and selective detection of mercury(II) ion. *Chem. Commun.* **46,** 1476-1478 (2010).
48. Wang, Q. et al. Fluorescence quenching of carbon nitride nanosheet through its interaction with DNA for versatile fluorescence sensing. *Anal. Chem.* **85,** 12182-12188 (2013).
49. Khani, H., Rofouei, M. K., Arab, P., Gupta, V. K. & Vafaei, Z. Multi-walled carbon nanotubes-ionic liquid-carbon paste electrode as a super selectivity sensor: application to potentiometric monitoring of mercury ion(II). *J. Hazard. Mater.* **183,** 402-409 (2010).
50. Li, M., Wang, Q., Shi, X., Hornak, L. A. & Wu, N. Detection of mercury(II) by quantum dot/DNA/gold nanoparticle ensemble based nanosensor via nanometal surface energy transfer. *Ana.l Chem.* **83,** 7061-7065 (2011).
51. Li, D., Wieckowska, A. &Willner, I. Optical analysis of Hg^{2+} ions by oligonucleotide-gold-nanoparticle hybrids and DNA-based machines. *Angew. Chem. Int. Ed. Engl.* **47,** 3927-3931 (2008).
52. Sahu, D., Sarkar, N., Mohapatra, P. & Swain, S. K. Rhodamine B

associated Ag/r-GO nanocomposites as ultrasensitive fluorescent sensor for Hg^{2+}. *Microchem. J.* **154,** 104577 (2020).

53. Ma, W.; Sun, M.; Xu, L.; Wang, L.; Kuang, H. & Xu, C., A SERS active gold nanostar dimer for mercury ion detection. *Chem. Commun. (Cambridge, U. K.)* **49,** 4989-4991 (2013).

54. Zhang, L. et al. Nanoporous Gold Based Optical Sensor for Sub-ppt Detection of Mercury Ions. *ACS Nano* **7,** 4595–4600 (2013).

55. Tan, H., Liu, B. & Chen, Y. Lanthanide Coordination Polymer Nanoparticles for Sensing of Mercury(II) by Photoinduced Electron Transfer. *ACS Nano* **6,** 10505-10511 (2012).

56. Liu, Q., Peng, J., Sun, L. & Li, F. High-Efficiency Upconversion Luminescent Sensing and Bioimaging of Hg(II) by Chromophoric Ruthenium Complex-Assembled Nanophosphors. *ACS Nano* **5,** 8040-8048 (2011).

57. Zhang, B., Meng, H., Wang, X., Chang, H. & Wei, W. Bio-dye sensitized detection of Hg^{2+} based GO-ZnO-CdS nanohybrids. *Sens. Actuators, B* **253,** 495-501 (2017).

58. Xuan, F. Luo, X. & Hsing, I. M. Conformation-dependent exonuclease III activity mediated by metal ions reshuffling on thymine-rich DNA duplexes for an ultrasensitive electrochemical method for Hg^{2+} detection. *Anal. Chem.* **85,** 4586-4593 (2013).

59. Wu, D. et al. Ultrasensitive electrochemical sensor for mercury(II) based on target-induced structure-switching DNA. *Biosens. Bioelectron.* **25,** 1025-1031 (2010).

60. Wu, C., Gao, G., Zhai, K., Xu, L. & Zhang, D. A visual Hg^{2+} detection strategy based on distance as readout by G-quadruplex DNAzyme on microfluidic paper. *Food Chem.* **331,** 127208 (2020).

61. Lin, Z., Li, X. & Kraatz, H. B. Impedimetric immobilized DNA-based sensor for simultaneous detection of Pb^{2+}, Ag^{+}, and Hg^{2+}. *Anal. Chem.* **83,** 6896-6901 (2011).

62. Li, T., Li, B., Wang, E. & Dong, S. G-quadruplex-based DNAzyme for

sensitive mercury detection with the naked eye. *Chem. Commun.* **24,** 3551-3553 (2009).

63. Lee, J., Jun, H. & Kim, J. Polydiacetylene-Liposome Microarrays for Selective and Sensitive Mercury(II) Detection. *Adv. Mater.* **21,** 3674-3677 (2009).

64. Hossain, S. M. & Brennan, J. D. beta-Galactosidase-based colorimetric paper sensor for determination of heavy metals. *Anal. Chem.* **83,** 8772-8778 (2011).

65. Cui, L., Wu, J., Li, M. & Ju, H. Highly sensitive electrochemical detection of mercury(II) via single ion-induced three-way junction of DNA. *Electrochem. Commun.* **59,** 77-80 (2015).

66. Huang, P. J., Wang, F. & Liu, J. Cleavable Molecular Beacon for Hg^{2+} Detection Based on Phosphorothioate RNA Modifications. *Anal. Chem.* **87,** 6890-6895 (2015).

67. Zhu, Y. et al. Highly sensitive colorimetric sensor for Hg^{2+} detection based on cationic polymer/DNA interaction. *Biosens. Bioelectron.* **69,** 174-178 (2015).

68. Liu, S. et al. Electrochemical DNA Biosensor Based on Microspheres of Cuprous Oxide and Nano-chitosan for Hg(II) Detection. *Electrochim. Acta* **160,** 64-73 (2015).

69. Jia, J. et al. A regenerative ratiometric electrochemical biosensor for selective detecting Hg^{2+} based on Y-shaped/hairpin DNA transformation. *Anal. Chim. Acta.* **908,** 95-101 (2016).

70. Huang, R. F., Liu, H. X., Gai, Q. Q., Liu, G. J. & Wei, Z. A facile and sensitive electrochemiluminescence biosensor for Hg^{2+} analysis based on a dual-function oligonucleotide probe. *Biosens. Bioelectron.* **71,** 194-199 (2015).

71. Ding, S. Y. et al. Thioether-Based Fluorescent Covalent Organic Framework for Selective Detection and Facile Removal of Mercury(II). *J. Am. Chem. Soc.* **138,** 3031-3037 (2016).

72. Cui, X. et al. A fluorescent biosensor based on carbon dots-labeled

oligodeoxyribonucleotide and graphene oxide for mercury (II) detection. *Biosens. Bioelectron.* **63,** 506-512 (2015).

73. Shahat, A., Elsalam, S. A., Herrero-Martínez, J. M., Simó-Alfonso, E. F. & Ramis-Ramos, G. Optical recognition and removal of Hg(II) using a new self-chemosensor based on a modified amino-functionalized Al-MOF. *Sens. Actuators, B* **253,** 164-172 (2017).

74. Li, P., Zhang, D., Jiang, C., Zong, X. & Cao, Y. Ultra-sensitive suspended atomically thin-layered black phosphorus mercury sensors. *Biosens. Bioelectron.* **98,** 68-75 (2017).
